# Quality of life in the older adults: The protective role of self-efficacy in adequate coping in patients with chronic diseases

**DOI:** 10.3389/fpsyg.2023.1106563

**Published:** 2023-04-06

**Authors:** Lorena Cudris-Torres, Stefano Vinaccia Alpi, Álvaro Barrios-Núñez, Natali Gaviria Arrieta, Jéssica Mejía Gutiérrez, Libia Alvis Barranco, Gerson Rios-Carlys, Silvia E. Cuenca-Calderón, Valmore Bermúdez, Juan Hernández-Lalinde, Carlos Arturo Riveira Zuleta, Marly Johana Bahamón, Juany Sofía Álvarez Herrera

**Affiliations:** ^1^Programa de Psicología, Fundación Universitaria del Área Andina, Valledupar, Colombia; ^2^Facultad de Ciencias de la Salud, Universidad del Sinú, Montería, Colombia; ^3^Clínica General del Norte, Barranquilla, Colombia; ^4^Programa de Psicología, Universidad Popular del Cesar, Valledupar, Colombia; ^5^Centro de Investigaciones en Ciencias de la Vida, Universidad Simón Bolívar, Barranquilla, Colombia; ^6^Universidad Simón Bolívar, Cúcuta, Colombia; ^7^Universidad del Sinú, Montería, Colombia

**Keywords:** well-being, mental health, skill, personality, emotions

## Abstract

The purpose of the present study was to establish the association between self-efficacy, perception of disease, emotional regulation, and fatigue and the health-related quality of life in older adults living in the departments of Cesar and Atlántico in Colombia and who have been diagnosed with a chronic disease. The participants were 325 older adults of both sexes, with literacy and no presence of cognitive impairment in the Mini-Mental State Examination (MMSE); A non-probabilistic sampling was carried out. We used the MOS-SF-36 questionnaire, the Brief Illness Perception Questionnaire scale for measuring the perception of disease, the Stanford Patient Education Research Center’s Chronic Disease Self self-efficacy questionnaire for chronic patients, the Difficulties in Emotional Regulation Scale, and the Fatigue Severity Questionnaire as measurement instruments. The design was non-experimental cross-sectional with a correlational scope. The results indicate that self-efficacy, disease perception, emotional regulation and severity of fatigue are variables that could impact the physical function of quality of life, confirming that self-efficacy would work as a factor that decreases the probability that a participant score low on this dimension of quality of life. On the other hand, both the perception of the disease and the severity of fatigue were identified as factors that probably negatively influence quality of life.

## 1. Introduction

Over the past 200 years, average human life expectancy has doubled in most developed and developing countries. Better water quality, nutrition, hygiene, housing, immunization against infectious diseases, antibiotics, and improved medical care reduced mortality for those in the early years of life and then after about 1950, among people in their 70s (Partridge et al., 2018; Mitchell and Walker, 2020). However, the increased longevity and owing to lifestyle changes may have led to a significant increase in the prevalence of noncommunicable diseases (NCDs), also known as chronic diseases—which are medical conditions associated with long duration and slow progression—in the human population. NCDs are non-infectious and are the result of interaction of several factors, including genetic, physiological, behavioral, social, and environmental factors (Martinengo et al., 2019). According to the World Health Organization (2013), NCDs are the leading cause of death worldwide, accounting for 71% of the total number of deaths per year.

The four leading causes of death among NCDs with the highest number of deaths are cardiovascular diseases (17.9 million deaths annually), cancer (9.0 million), respiratory diseases (3.9 million), and diabetes (1.6 million) (World Health Organization, 2019, 2020). Many individuals with chronic diseases do not know how to cope with their disease. They have enormous difficulties in understanding its causes and do not know how to monitor it, i.e., they do not know how the disease should be managed. This leads to deterioration of their well-being and quality of life and emerging negative emotions including anxiety, depression, hopelessness, and stress. This results in inadequate monitoring of their disease and as a consequence over time, to the appearance of episodes of learned hopelessness (Bica et al., 2017). On the contrary, positive self-management of the disease can be associated with self-efficacy which can be defined as an individual’s belief of their ability to control adverse events that may influence their life (Dunke Pereira et al., 2018).

Self-efficacy plays an important role in determining whether actions of self-care are initiated, how much effort is expended, and how long the effort is sustained in the presence of obstacles and failures (Bandura and Freeman, 1997). Patients that have a high level of self-efficacy when faced with their chronic diseases show a perceived ability to cope with the challenges related to their diseases and a sense of control over their lives (Bandura and Freeman, 1997; Cheng et al., 2019; Chan, 2021). In this regard, empirical studies have been developed in the last 20 years based on the favorable influence of self-efficacy in the management of chronic diseases. The diseases include chronic obstructive pulmonary disease (Kar and Zengin, 2020; Selzler et al., 2020; Paneri and Napolitano, 2021; Yi et al., 2021), chronic renal failure (Brioni et al., 2021), chronic diseases in general (Almutary and Tayyib, 2020; Farley, 2020; Yuan et al., 2021), and stroke (Bailey, 2019).

However, in recent years a number of studies have focused on understanding how relevant the mental representation of the disease is in the patient’s perception of the disease. For example, owing to negative emotions, patients perceive that the duration of the disease is much longer and the consequences to be more severe while greater personal and treatment control corresponds to a less severe perception of the consequences of the disease. This is observed in chronic diseases in general (Özkan Tuncay et al., 2018; Cheng et al., 2020; Giuffrida et al., 2021; Szabó-Bartha and Mirnics, 2021) and cases of chronic renal failure (Dantas et al., 2020), multiple sclerosis (Timkova et al., 2021), hepatitis B and C (Ekmen et al., 2021), leukemia (Arrato et al., 2022), and systemic lupus erythematosus (Elefante et al., 2020).

Similarly, some studies refer to the role that fatigue plays in the perception of well-being and quality of life of the chronically ill patients. Fatigue can be defined as the subjective feeling of a lack of physical and mental energy, perceived by a person, which interferes with daily activities (Gregg et al., 2021). Fatigue is a disabling symptom, which often covaries with depressive symptoms, anxiety, and sleep impairment and it is associated with a poor state of subjective health perception and a low level of quality of life (Adamowicz et al., 2022). The studies include those on chronic disease in general (Menting et al., 2018; Torossian and Jacelon, 2021), irritable colon (Nocerino et al., 2020), and renal failure (Swain and Jones, 2020; Gregg et al., 2021); similarly, numerous studies have referred to the impact of different chronic diseases on health-related quality of life (HRQOL).

HRQOL is a multidimensional construct consisting of at least three broad domains (physical, psychological, and social functioning) that are affected by the disease itself and/or its treatment. The associated conditions include chronic low back pain (Járomi et al., 2021), chronic venous insufficiency (Silva et al., 2021), systemic lupus erythematosus (Elefante et al., 2020), renal transplantation (Sawada et al., 2021), globus pharyngeus (Zhang and Liu, 2021), Lyme disease (Hill and Frost, 2022), and chronic respiratory failure (Oga et al., 2018).

Similarly, emotional regulation, i.e., experiencing, processing, and modulating emotional responses, is necessary to manage common emotional stressors in patients with chronic disease. Overwhelming emotional demands deplete the resources needed for the daily management of chronic disease self-care and they contribute to poor health outcomes (Wierenga et al., 2017, 2018; Zarotti et al., 2018; Della Vecchia et al., 2019). Pertinent studies in the past have focused on cases of cardiovascular disease (Roy et al., 2018; Luque et al., 2020) and chronic disease in general (Bramanti et al., 2021).

In spite of the epidemiological projections on the high prevalence of chronic diseases in Colombia (Gallardo-Solarte et al., 2016) and considering the impact of self-efficacy, disease perception, self-regulation, and fatigue on HRQOL, several studies have focused on this subject worldwide. In addition, there is a paucity of empirical studies in Colombia from the field of health (Ardila, 2018). Therefore, the aim of this study was to analyze and compare the relationship that self-efficacy, illness perception, self-regulation, fatigue, and socio-demographic and clinical characteristics have on HRQOL in Colombian patients with chronic diseases.

## 2. Materials and methods

### 2.1. Design

This is a quantitative non-experimental, cross-sectional research with a correlational scope and a survey-type data collection model (Hernández et al., 2014).

### 2.2. Participants

Non-random sample of 325 older adults with a medical diagnosis of a chronic disease of at least 1 year with a mean age and standard deviation of 68.39 and 4.15 years (CV = 6.07%), respectively. Residents of the departments of Cesar and Atlántico, participating voluntarily and provided informed consent. Subjects had no cognitive impairment (Mini-mental State Examination [MMSE] scores of >23) and had reading and writing ability so that they could understand the tests.

The conditions of the population of interest made it impossible to obtain a sampling frame, forcing the researchers to select the participants through a non-probabilistic convenience sampling. Given this situation, the sample size was determined based on the maximum number of participants that could be selected within the limitations of the research.

Therefore, the study included 390 participants, of whom 65 presented cognitive impairment in the application of the Mini-Mental State Examination (MMSE), this being an exclusion criterion; The final response rate was 83.33%.

### 2.3. Instruments

The mini-mental scale is a brief psychometric scale to evaluate the cognitive state of people and the Colombian version of this scale has been introduced by Rosselli et al. (2000). This scale was introduced into clinical practice as a practical method to assess the cognitive status of their patients. This instrument is composed of 30 dichotomous items that evaluate six cognitive processes: temporal orientation, spatial orientation, fixation memory, recall memory, attention and calculation, and language; the internal consistency is adequate (Cronbach’s alpha: 0.807).

The Fatigue Intensity scale (FSS) consists of nine items with a Likert-type response of seven possibilities of increasing intensity and scores between 1 and 7. The total is the sum of all the items. The Colombian version of Riveros Munévar et al. (2017) was used for this study.

The Colombian version of the Difficulties in Emotion Regulation Scale (DERS) was prepared by Muñoz-Martínez et al. (2006). This measures emotional dysregulation and consists of 36 items grouped into six factors as follows: (a) non-acceptance of emotional responses (non-acceptance), (b) difficulties in goal-directed behaviors when upset (goals), (c) difficulties in controlling impulsive behaviors when upset (impulsivity), (d) limited access to emotional regulation strategies that are perceived as effective (strategies), (e) lack of emotional awareness (awareness), and (f) lack of emotional clarity (clarity). The analysis of the DERS applied in a sample of Colombians revealed an internal consistency *α* = 0.90.

The Stanford Patient Education Research Center’s Chronic Disease Self-Efficacy (SEMCD-S) was developed by Ritter and Lorig (2014). This consists of four items on a scale of 1–10, with 1 being “Very Insecure” and 10 being “Very Confident.” This is an instrument for measuring the self-efficacy related to chronic disease management. Research reports a reliability of 0.95 (22) and for this research a Cronbach’s alpha of *α* = 0.791 was obtained.

MOS SF-36 Health Questionnaire was validated in Colombia by Lugo et al. (2006). This questionnaire consists of 36 items that explore eight dimensions of health status corresponding to physical function, social function, role limitations: physical problems, emotional problems, mental health, vitality, pain, and perception of general health. The reliability of the MOSSF-36 Health Questionnaire is 0.7; the physical role, physical function, and emotional role measurements obtained reliability scores equal to 0.90; in terms of content validity they had high correlations with the physical component (*r* ≥ 0.74).

Brief Illness Perception Questionnaire (Brief Illness Perception Questionnaire [IPQ-B]). In the Spanish version of Pacheco-Huergo et al. (2012), this questionnaire consists of nine items with Likert-type responses ranging from 0 to 10 points. It comprises eight dimensions and an ordinal open-ended question with three possible answers. These dimensions are as follows: consequences, duration, personal management, treatment management, identity, worry, emotional response and understanding of the disease. Item number 9 is an open-ended question in reference to the factors that caused his or her disease according to the person and organized accordingly of their importance. The questionnaire has adequate internal consistency indices ranging from *α* = 0.67–0.89.

### 2.4. Procedure

Before starting the field phase of the research, which included the instrument application, 16 adults aged >60 years were selected and they underwent a pilot test to identify the words or questions that were difficult to understand to identify the respective comprehension in the response system and to establish the time for each of the instruments. At the end of this process, there was clarity and understanding on the part of the participants of the pilot test.

A total of 325 older adults were selected from the departments of Cesar and Atlántico based on the fact that they were older than 60 years, met the characteristics of having a diagnosis of a chronic disease, and voluntarily decided to participate.

At the outset, a brief explanation of the research was performed, explaining the objectives and procedures to be performed. After this, informed consents were signed, so that there was a written agreement with the participants to authorize their voluntary participation; This was followed by the scaling of sociodemographic data in order to find out the characteristics of the population and with these data, the MMSE was applied, which helped to rule out the subjects who presented cognitive impairment, ruling out cognitive impairment in the participants, this was followed by applying the instruments in the following order (FSS, DERS, SEMCD-S, MOS SF-36 and IPQ-B), the whole process lasted approximately 2 h per participant. The data obtained were incorporated into a database and analyzed using the SPSS-27 statistical package.

### 2.5. Analysis plan

Firstly, an exploratory data analysis (EDA) was conducted in order to evaluate compliance with the assumptions needed to perform parametric tests, as well as to assess whether the use of multiple linear regression (MLR) was an appropriate option. In this regard, the normality of the variables was tested by means of the Shapiro–Wilk (SW) test, also opting for the construction of Q-Q plots as a complementary tool. Equality of variances was examined using Levene’s test, considering each of the sociodemographic characteristics as grouping factors. Independence of observations was inspected at statistical level using the median-based Wald-Wolfowitz cluster test, while the MLR conditions were validated by means of a thorough analysis of the residuals of each model.

In this regard, SW contrast was used again to check whether the residuals conformed to a normal distribution, while homoscedasticity was inspected graphically and analytically through typed scatter plots and by means of the Breusch–Pagan (BP) and White procedures. The residual autocorrelation assumption was evaluated with the Durbin-Watson (DW) statistic, while the presence of multicollinearity was checked using the variance inflation factors (VIF) and the condition index (CI). After this preliminary phase, it was concluded that nonparametric tests and regression options involving less demanding statistical assumptions should be used due to the violation of the aforementioned assumptions.

Once these conjectures were discarded, the research variables were described using basic summary measures. The raw or transformed scores were characterized through the minimum, maximum, range, interquartile range, mean, median, mode, variance, standard deviation, coefficient of variation, lower quartile, and upper quartile.

Furthermore, the sociodemographic characteristics compiled in the study, most of which were attribute variables, were presented in the form of absolute and relative frequencies. To compare the effect of these factors on the variables of interest, the Mann–Whitney U test (MWU) was selected when these characteristics consisted of two groups, choosing the Kruskal–Wallis H test (KWH) when, on the contrary, the number of levels was greater than or equal to three. On this occasion, the *post hoc* contrasts were conducted with the MWU method; however, the outcomes were corrected for significance *via* Bonferroni correction.

The dependent variables of the binary logistic regression (BLR) models were set from each of the eight dimensions of quality of life and used as predictors of the scores of the global constructs of self-efficacy, perception of disease, emotional regulation, and fatigue severity. The study was advanced in this way and each dimension was not incorporated as a regressor owing to the multicollinearity found between the factors of each scale, which had a negative impact on the VIF and the CI, increasing their values considerably.

In addition to the abovementioned statistical argument, there are numerous empirical and theoretical evidences to justify the use of global constructs of these instruments for description, classification, and prediction purposes. However, the categorization of the DVs was obtained by defining those scores below the lower quartile and above the upper quartile as low and high levels, which enabled the dichotomous characteristics necessary for the use of the BLR to be obtained. In addition, age, sex, time of disease diagnosis and the patient’s circumstances with respect to whether he/she lives alone or accompanied were introduced to the regression models to obtain adjusted estimates and odds ratios that truly reflected the magnitude of the association between the variables. Linearity between the predictors of the BLR and the logarithm of the odds ratio was checked through the Box-Tidwell procedure without finding relevant inconsistencies, while the existence of outliers and influence points was inspected by means of residuals, Cook’s and Mahalanobis distances. The presence of certain anomalies or influence data was moderate and did not impact the estimates.

Finally, data processing and analysis were performed with the IBM SPSS statistical package (version 27 for Windows 64-bit). Results whose significance was less than 0.05, 0.01 or 0.001 were considered significant.

## 3. Results

### 3.1. Sociodemographic characteristics of the participants

The sample consisted of 325 people, of which 46.46% (*n* = 151) were female and 53.54% (*n* = 174) were male. Their age ranged from 60 to 78 years, with a mean of 68.39 and standard deviation of 4.15 (CV = 6.07%). In terms of time since disease diagnosis, values ranged from 5 to 60 years, with a mean of 15.79 and variability of 6.96 (CV = 44.08%). Other participant characteristics, such as the municipality of residence, marital status, whether living alone or accompanied, education, socioeconomic status, health system, psychiatric medication intake and treatment for their chronic condition are shown in Table 1.

**Table1:** Sociodemographic characteristics of the participants.

Variable	Categories	*n*	%
Sex	Female	151	46.46
	Male	174	53.54
Municipality	Valledupar	133	40.92
	Chimichagua	73	22.46
	Barranquilla	119	36.62
Marital status	Single	62	19.08
	Married	145	44.62
	Separated or divorced	4	1.23
	Cohabiting	99	30.46
	Widowed	15	4.62
Who do you live with?	Alone	46	14.15
	In the company of family members	271	83.38
	In the company of friends	8	2.46
Education	Completed elementary school	80	24.62
	Incomplete elementary school	91	28.00
	Incomplete baccalaureate	61	18.77
	Completed bachelor’s degree	77	23.69
	University	16	4.92
	Postgraduate	0	0.00
Socioeconomic stratum	Stratum 1	169	52.00
	Stratum 2	148	45.54
	Stratum 3	8	2.46
	Stratum 4	0	0.00
	Stratum 5	0	0.00
	Stratum 6	0	0.00
Health system	Subsidized	310	95.38
	Contributory	15	4.62
	Exempt	0	0.00
	Prepaid	0	0.00
Do you take medication for psychiatric problems?	Yes	12	3.69
	No	313	96.31
Do you take medication for your chronic disease?	Yes	325	100.00
	No	0	0.00

Compiled by authors.

Quality of life, self-efficacy, illness perception, emotional regulation, and fatigue.

The physical function of quality of life will be used as an example to develop this section. As can be seen in Table 2, it was found that the scores in this dimension ranged from 20 to 80 points, involving a range of 60 points. The lower and upper quartiles were 30 and 70, respectively, thus obtaining an interquartile range of 40 points. Regarding the centralization measurements, the scores averaged 49.91 points, with a median of 50 and a mode of 30. Variability was observed around the mean of 21.42 points, thus reflecting a relatively high coefficient of variation of 42.92%. For practicality, the characterization of the rest of the variables is omitted and the reader is referred to the detailed information shown in Table 2.

**Table 2:** Raw or transformed scores of the variables analyzed in the research.

Variables	Min.	Q_1_	Me	Q_3_	Max.	*R*	IQR	*M*	Mo	DE	CV
*Self-efficacy*	4	21	24	25	35	31	4	23.28	22	6.10	26.20
*Disease perception*	40	47	51	58	61	21	11	51.69	60	6.43	12.44
Consequences	4	8	8	10	10	6	2	8.46	10	1.53	18.09
Duration	6	10	10	10	10	4	0	9.60	10	1.01	10.52
Personal control	0	3	5	6	9	9	3	4.72	6	2.03	43.01
Treatment control	2	2	3	4	9	7	2	3.23	3	1.44	44.58
Identity	6	7	8	10	10	4	3	8.32	8	1.29	15.50
Concern	4	8	9	10	10	6	2	8.52	10	1.56	18.31
Understanding the disease	0	0	0	1	6	6	1	0.79	0	1.61	203.80
Emotional response	3	7	8	10	10	7	3	8.05	10	1.74	21.61
*Emotional regulation*	10	37	44	58	68	58	21	41.26	10	19.54	47.36
Cognitive regulation	6	30	35	36	42	36	6	28.59	36	12.97	45.37
Emotional suppression	4	4	8	22	28	24	18	12.68	4	9.15	72.16
*Severity of fatigue*	9	34	40	63	63	54	29	43.41	63	16.57	38.17
Motivational impairment	1	5	5	7	7	6	2	5.63	5	1.46	25.93
Physical involvement	5	16	24	35	35	30	19	24.25	35	9.64	39.75
Social impact	3	9	12	21	21	18	12	13.53	21	6.19	45.75
*Quality of life*	NA	NA	NA	NA	NA	NA	NA	NA	NA	NA	NA
Physical function	20	30	50	70	80	60	40	49.91	30	21.42	42.92
Physical role	0	0	100	100	100	100	100	53.85	100	48.99	90.97
Body pain	0	12	52	74	100	100	62	45.22	0	34.68	76.69
General health	5	20	40	45	77	72	25	35.95	45	18.05	50.21
Vitality	0	25	50	80	90	90	55	55.06	20	27.76	50.42
Social function	25	50	50	50	75	50	0	51.07	50	11.16	21.85
Emotional role	0	0	100	100	100	100	100	52.60	100	48.98	93.12
Mental health	8	44	72	92	96	88	48	66.81	44	24.56	36.76

Compiled by authors. Min, minimum; Q_1_, lower quartile; Me, median; Q_3_, upper quartile; Max, maximum; R, range; IQR, interquartile range; M, mean; Mo, mode; SD, standard deviation; CV, coefficient of variation; NA, not applicable.

### 3.2. Comparison by sociodemographic characteristics

Tables 3–5 show the results of assessing the effect of the sociodemographic characteristics on the variables of interest for the study. It is emphasized at this point what was clarified in the statistical analysis section: nonparametric tests were used as a consequence of the non-observance of the postulates under which these tests operate. Thus, the data contrasted in these tables respond to the average assigned ranges, this is a typical transformation applied in these tests and is used to carry out the procedure. Therefore, special care must be taken when interpreting these values since they do not correspond to the raw scores.

**Table 3:** Self-efficacy and perception of disease according to sociodemographic characteristics.

Variables	AET	PE1	PE2	PE3	PE4	PE5	PE6	PE7	PE8	PET
Female	165.50a	169.18a	159.14ª	160.82a	171.62a	166.50a	170.47a	167.77a	167.77a	172.47a
Male	160.83a	157.64a	166.35ª	164.89a	155.52a	159.96a	156.51a	158.86a	158.86a	154.78a
Taking psychiatric medication	212.00a	124.50a	190.00a	109.83a	49.00a	61.00a	70.50a	121.50a	44.67a	35.00a
Does not take medicines	161.12a	164.48a	161.96ª	165.04b	167.37b	166.91b	166.55b	164.59b	167.54b	167.91b
Contributory health system	156.07a	109.57a	190.00a	130.20a	100.13a	176.13a	160.30a	121.50a	142.17a	134.10a
Subsidized health system	163.34a	165.59b	161.69ª	164.59a	166.04b	162.36a	163.13a	165.01b	164.01a	164.40a
Lives accompanied	154.65a	167.27a	161.79ª	161.49a	171.82a	171.60a	170.13a	159.35a	167.32a	170.58a
Lives alone	213.63b	137.13b	170.33ª	172.14a	109.50b	110.83b	119.73b	185.14b	136.83b	117.02b
Stratum 2 or 3	150.04a	160.04a	171.76ª	165.14a	161.71a	172.13a	170.51a	148.63a	172.40a	168.34a
Stratum 1	174.96b	165.73a	154.91b	161.02a	164.19a	154.57a	156.07a	176.26b	154.32a	158.07a
Valledupar	150.12a	182.00a	175.80ª	177.11a	173.33a	178.80a	175.59a	150.62a	171.79a	180.33a
Chimichagua	161.90a	175.86a	171.29ª	171.00a	166.40a	170.04a	168.61a	158.51a	165.85a	171.55a
Barranquilla	178.08a	133.87b	143.61b	142.32b	149.37a	141.02b	145.49b	179.59b	151.43a	138.38b
Married or cohabiting	159.83a	158.23a	161.05a	156.90a	159.90a	166.30a	162.24a	161.30a	167.41a	162.24a
Single, separated or divorced	193.18b	157.24a	164.07a	169.35a	158.83a	134.46b	154.33a	167.34a	137.27a	144.15b
Widowed	81.80c	266.00b	190.00a	234.23b	231.83b	234.83c	213.50a	171.50a	204.50b	258.33a
University or higher education	41.13a	195.25a	190.00a	173.13a	159.38a,b	170.00a	166.75a	121.50a	94.37b	164.50a
Baccalaureate	168.54b	171.61a	153.26ª	162.18a	179.74a	170.22a	168.90a	172.70b	170.35a	172.95a
Primary	169.93b	153.04a	168.33ª	162.72a	149.83b	156.51a	157.89a	159.05a,b	163.49a	154.83a

Compiled by authors. AET, self-efficacy (global construct); PE1, consequences; PE2, duration; PE3, personal control; PE4, treatment control; PE5, identity; PE6, worry; PE7, understanding of the disease; PE8, emotional response; PET, disease perception (global construct).

The average assigned ranks are shown. In addition, those average assigned ranks that share the same sub-index do not differ statistically at the 0.05 level corrected according to the Bonferroni procedure when this condition applies (three or more groups). To make comparisons, variables such as socioeconomic stratum, marital status and education underwent a grouping of their categories because very few counts were observed in some of them (see Table 1). The intensity of the gray shading indicates the magnitude of the observed value of *p*: light, *p* < 0.05; medium, *p* < 0.01: dark, *p* < 0.001.

**Table 4:** Emotional regulation and fatigue severity according to sociodemographic characteristics.

Variables	RE1	RE2	RET	SF1	SF2	SF3	SFT
Female	168.01a	163.29a	164.92a	160.22a	170.42a	167.83a	169.44a
Male	158.66a	162.75a	161.34a	165.42a	156.56a	158.81a	157.41a
Taking psychiatric medication	205.33a	260.17a	255.33a	101.50a	42.17a	27.33a	32.67a
Does not take medicines	161.38a	159.27b	159.46b	165.36b	167.63b	168.20b	168.00b
Contributory health system	156.83a	174.07a	172.00a	133.40a	151.67a	126.17a	132.60a
Subsidized health system	163.30a	162.46a	162.56a	164.43a	163.55a	164.78a	164.47a
Lives accompanied	151.44a	154.21a	153.73a	170.91a	174.69a	174.13a	174.96a
Lives alone	233.12b	216.29b	219.22b	115.04b	92.08b	95.47b	90.47b
Stratum 2 or 3	151.99a	146.62a	147.96a	172.23a	178.71a	175.03a	176.91a
Stratum 1	173.17b	178.12b	176.89b	154.48a	148.50b	151.90b	150.16b
Valledupar	153.97a	155.30a	155.00a	173.23a	172.35a	173.51a	174.06a
Chimichagua	169.16a	165.84a	167.17a	161.16a	161.63a	163.85a	162.90a
Barranquilla	169.31a	169.86a	169.39a	152.69a	153.39a	150.73a	150.70a
Married or cohabiting	153.18a	156.35a,b	154.60a,b	169.66a	168.32a	168.51a	169.26a
Single, separated or divorced	212.89a	193.62a	202.01a	121.98b	124.17b	126.48b	120.77b
Widowed	103.27b	136.50b	127.93b	235.17c	247.33c	234.00c	247.00c
University or higher education	136.50a	166.13a	176.12a	181.25a	157.13a	162.00a	163.63a
Baccalaureate	168.80a	159.42a	162.64a	156.03a	168.97a	174.03a	171.16a
Primary	160.80a	165.60a	162.06a	166.92a	158.73a	154.19a	156.36a

Compiled by author. RE1, cognitive regulation; ER2, emotional suppression; RET, emotional regulation (global construct); SF1, motivational impairment; SF2, physical affect; SF3, social impairment; SFT, fatigue severity (global construct).

The average assigned ranks are shown. In addition, those average assigned ranks that share the same sub-index do not differ statistically at the 0.05 level corrected according to the Bonferroni procedure when this condition applies (three or more groups). To make comparisons, variables such as socioeconomic stratum, marital status and education underwent a grouping of their categories because very few counts were observed in some of them (see Table 1). The intensity of the gray shading indicates the magnitude of the observed value of *p*: light, *p* < 0.05; medium, *p* < 0.01: dark, *p* < 0.001.

**Table 5:** Quality of life according to sociodemographic characteristics.

Variables	CV1	CV2	CV3	CV4	CV5	CV6	CV7	CV8
Female	153.68a	168.60ª	158.23a	171.46a	168.27a	164.15a	168.58a	167.24a
Male	171.09a	158.14ª	167.14a	155.66a	158.43a	162.01a	158.16a	159.33a
Taking psychiatric medication	302.00a	244.00a	40.50a	168.17a	287.67a	58.50a	242.50a	265.00a
Does not take medicines	157.67b	159.90b	167.70b	162.80a	158.22b	167.01b	159.95b	159.09b
Contributory health system	176.93a	198.53ª	185.23a	200.70a	196.63a	150.30a	197.17a	188.80a
Subsidized health system	162.33a	161.28ª	161.92a	161.18a	161.37a	163.62a	161.35a	161.75a
Lives accompanied	157.57a	155.15ª	171.28a	161.02a	154.44a	163.78a	155.17a	151.14a
Lives alone	195.92b	210.64b	112.80b	175.01a	214.91b	158.29a	210.49b	234.96b
Stratum 2 or 3	156.00a	154.11ª	177.33a	159.08a	150.89a	165.31a	154.02a	150.46a
Stratum 1	169.46a	171.21ª	149.77a	166.62a	174.18b	160.87a	171.29a	174.58b
Valledupar	159.99a	152.73ª	165.18a	158.40a	154.34a	162.71a	151.50a	150.45a
Chimichagua	165.79a	162.02ª	159.21a	166.03a	162.88a	164.76a	163.02a	161.36a
Barranquilla	164.65a	175.08ª	162.89a	166.28a	172.75a	162.25a	175.84a	178.03a
Married or cohabiting	156.53a	160.93ª	163.35a	165.63a	157.47a,b	166.48a	158.90a	159.50a,b
Single, separated or divorced	210.16b	178.06ª	150.98a	165.23a	196.44b	151.17a	185.83a	188.65b
Widowed	60.83c	130.33ª	210.20a	110.43a	105.80a	158.50a	129.17a	107.07a
University or higher education	237.75a	116.13ª	160.75a	193.75a	160.13a	165.50a	115.00a	90.75a
Baccalaureate	158.24b	158.38a,b	166.86a	169.42a	154.33a	172.47a	160.88b	152.86b
Primary	159.85b	171.11b	160.10a	154.94a	170.27a	155.12a	169.21b	177.95b

Compiled by authors. CV1, physical function; CV2, physical role; CV3, bodily pain; CV4, general health; CV5, vitality; CV6, social role; CV7, emotional role; CV8, mental health.

The average assigned ranks are shown. In addition, those average assigned ranks that share the same sub-index do not differ statistically at the 0.05 level corrected according to the Bonferroni procedure when this condition applies (three or more groups). To make comparisons, variables such as socioeconomic stratum, marital status and education underwent a grouping of their categories because very few counts were observed in some of them (see Table 1). The intensity of the gray shading indicates the magnitude of the observed value of *p*: light, *p* < 0.05; medium, *p* < 0.01: dark, *p* < 0.001.

Having said this and taking as a reference whether the patient takes psychiatric medication to cope with his or her condition, it was observed that this factor had an effect on the scores of the “personal control” dimension of the disease perception scale. It is noted that those not taking these medications reported higher assigned ranges than those who do (165.04 vs. 109.83, *p* < 0.05), which could be interpreted as meaning that the perception of disease is higher among those subjects who do not have the benefit of this palliative. When analyzing the health system and its influence, it can be seen that the participants belonging to the contributory system exhibit lower ratings in the “consequences” aspect of this questionnaire than those in the subsidized regime (109.57 vs. 165.59, *p* < 0.05), implying that the repercussions of the disease are conceived to be less serious among these individuals. However, it is important to emphasize that previous results should be interpreted cautiously because the sample size in one group is small and very different from that in the other group. As such, the validity of these findings may be questionable.

Regarding the severity of fatigue, it is noted that those using psychiatric medication reached significantly lower figures than those not doing so, this was observed in the dimensions of motivational affectation (101.50 vs. 165.36, *p* < 0.01), physical (92.08 vs. 174.69, *p* < 0.001), social (95.47 vs. 174.13, *p* < 0.001) and in the questionnaire total (90.47 vs. 174.96, *p* < 0.001).

As regards quality of life, it was found that marital status had a significant effect on the patients’ assessment of this construct. Specifically, widowers reported much lower ratings in the aspect of this variable defined as “physical function” than those who were single, separated or divorced (60.83 vs. 210.16, *p* < 0.001), but also than those who were married or cohabiting (60.83 vs. 156.53, *p* < 0.001). In turn, participants cohabiting with their partner reflected lower assigned ranks than those who were separated or single (156.53 vs. 210.16, *p* < 0.001). A similar situation was observed in vitality and mental health, both subscales of the MOS SF-36 instrument that measures health-related quality of life.

In an effort to avoid an excessive description of the results of this phase, the reader is encouraged to continue with this analysis by carefully reviewing the findings shown in Tables 3–5. In this regard, it should be noted that the APA methodology has been used for identifying whether two or more assigned ranges differ statistically, a procedure that involves assigning different letters as subscripts (a, b, c, etc.,). Furthermore, we have chosen to facilitate the visualization of the table by shading in gray those comparisons that were found to be significant, increasing the intensity of the shading as this property increases. Specifically: if highlighted in light gray, the value of p obtained is less than 0.05; if in medium gray, the significance observed is less than 0.01; and if in dark gray, the difference is statistically significant for values less than 0.001.

### 3.3. Association between quality of life, self-efficacy, disease perception, regulation, and fatigue

As noted in the statistical analysis section, when establishing the association between the aspects of quality of life (DV: dependent variables) and self-efficacy, disease perception, emotional regulation, and fatigue severity (IV: independent variables), BLR models were used. DVs were categorized from the lower and upper quartiles, while IVs were entered unmodified, in their raw or direct form. To obtain adjusted odds ratios (OR), covariates such as sex, age, disease duration and the patient’s living situation were incorporated into each model. The fit was assessed with Cox-Snell and Nagelkerke coefficients (pseudo R2), while the precision of the estimates was identified by displaying the bilateral confidence interval of the ORs together with their level of significance. All BLR assumptions were tested without any major irregularities or deviations being discovered.

Thus, the results indicate that self-efficacy, disease perception, emotional regulation, and severity of fatigue are variables that could impact physical function and quality of life. Note that each of these regressors reflected statistically significant odds ratios. Specifically, the perception of the disease and the severity of fatigue were identified as factors that probably negatively influence quality of life. It is noted that, if all other factors are held constant, for each point that self-efficacy increases, the probability of this happening is reduced by approximately 12% (OR = 0.88, *p* = 0.002, 0.81–0.95), a fact also observed under similar conditions, although less marked, in emotional regulation. In this case, if the other variables are not modified, a unit increase in this construct implies a 3% decrease (OR = 0.97, *p* = 0.027, 0.95–0.99) in the possibility of obtaining low levels of physical function. Conversely, both disease perception and fatigue severity were identified as risk variables. Regarding the former, one-point increases would lead to a 22% increase (OR = 1.22, *p* < 0.001, 1.11–1.34) in the probability of achieving low ratings in physical function; while, regarding the latter, unit increases in the fatigue severity scale would generate an increase in this risk of 13% (OR = 1.13, *p* < 0.001, 1.08–1.18). Judging from the pseudo R2s, about 57 to 77% of the variation in physical function would be explained by the model. To understand all the findings of this stage of the research, let us perform a similar description as in the previous paragraph with the rest of the models shown in Table 6.

**Table 6:** Relationship between quality of life, self-efficacy, perception of disease, emotional regulation, and severity of fatigue.

Variables	OR (*p,* LCI, LCS)	CS-R^2^, N-R^2^	Variables	OR (*p,* LCI, LCS)	CS-R^2^, N-R^2^
*Physical function (success = low score)*			*Vitality (success = low score)*		
Self-efficacy	0.88 (0.002, 0.81, 0.95)^*^	0.57, 0.77	Self-efficacy	1.01 (0.855, 0.90, 1.13) ^NS^	0.60, 0.83
Disease perception	1.22 (<0.001, 1.11, 1.34)^†^		Disease perception	1.14 (0.012, 1.03, 1.27)^†^	
Emotional regulation	0.97 (0.027, 0.95, 0.99)^*^		Emotional regulation	0.89 (<0.001, 0.85, 0.93)^*^	
Severity of fatigue	1.13 (<0.001, 1.08, 1.18)^†^		Severity of fatigue	1.09 (0.005, 1.03, 1.16)^†^	
*Physical role (success = low score)*			*Social function (success = low score)*		
Self-efficacy	0.49 (<0.001, 0.40, 0.61)^*^	0.58, 0.78	Self-efficacy	0.87 (<0.001, 0.81, 0.94) ^*^	0.13, 0.21
Disease perception	1.11 (0.020, 1.02, 1.22)^†^		Disease perception	1.12 (0.004, 1.04, 1.20)^†^	
Emotional regulation	0.75 (<0.001, 0.69, 0.82)^*^		Emotional regulation	0.98 (0.039, 0.96, 0.99)^*^	
Severity of fatigue	0.75 (<0.001, 0.67, 0.83)^*^		Severity of fatigue	0.97 (0.062, 0.96, 1.01)^NS^	
*Body pain (success = low score)*			*Emotional role (success = low score)*		
Self-efficacy	0.95 (0.161, 0.90, 1.02) ^NS^	0.27, 0.38	Self-efficacy	0.66 (<0.001, 0.54, 0.80) ^*^	0.63, 0.84
Disease perception	1.10 (0.012, 1.02, 1.19)^†^		Disease perception	1.11 (0.034, 1.01, 1.23)^†^	
Emotional regulation	1.02 (0.126, 0.99, 1.04)^NS^		Emotional regulation	1.04 (0.042, 1.00, 1.07)^†^	
Severity of fatigue	0.90 (<0.001, 0.87, 0.93)^*^		Severity of fatigue	1.28 (<0.001, 1.17, 1.41)^†^	
*General health (success = low score)*			*Mental health (success = low score)*		
Self-efficacy	1.00 (0.942, 0.89, 1.11)^NS^	0.62, 0.87	Self-efficacy	0.60 (<0.001 0.48, 0.75)^*^	0.67, 0.92
Disease perception	1.14 (0.029, 1.01, 1.27)^†^		Disease perception	1.70 (<0.001, 1.34, 2.17)^†^	
Emotional regulation	0.91 (<0.001, 0.86, 0.95)^*^		Emotional regulation	0.92 (0.038, 0.85, 0.99)^*^	
Severity of fatigue	1.13 (0.001, 1.05, 1.21)^†^		Severity of fatigue	1.09 (0.212, 0.95, 1.24)^NS^	

Compiled by the authors. OR, odds ratio or odds ratio; *p*, value of *p* or observed significance; LCI, lower 95% confidence limit; UCL, 95% upper confidence limit; CS-R^2^, Cox-Snell pseudo *R*^2^; N-R^2^, pseudo *R*^2^ of Nagelkerke; ^*^CS-R, statistically significant protective factor; ^†^CS-R, statistically significant risk factor; ^NS^N-R, nonsignificant factors.

“success” in the logistic regression model is defined as “participant exhibits a low quality of life score.” However, adjusted ORs are shown according to sociodemographic variables including age, time since diagnosis of the disease, sex, and whether the participant lives alone or accompanied. The dependent variables of each model were the quality-of-life aspects, while the independent variables were the global constructs of the self-efficacy, disease perception, emotional regulation and fatigue severity scales. The latter was done to overcome existing multicollinearity problems due to the high interfactorial correlation of each scale (see section on statistical analysis).

## 4. Discussion

The aim of this research was to establish the presence of associations between variables such as self-efficacy, perception of disease, emotional regulation, and fatigue and the health-related quality of life in older adults diagnosed with a chronic disease. The results demonstrated that it was possible to identify the existence of different associations between the variables analyzed.

It was found that older adults who had pharmacological treatment with psychiatric medication offered lower scores in personal control and a greater perception of disease. Therefore, it is important to consider that personal control relates to the beliefs that the individual has about monitoring the disease and their state of health and these are attributed to internal or external factors. In this sense, the literature has shown that this perception relates to different aspects, within these are the socioeconomic conditions of the individual and better levels of mental health and subjective well-being (April et al., 2012).

This result on the exposure to psychiatric pharmacological treatment would indicate the need to address aspects relating to the patient’s education concerning their health condition, in order to develop beliefs focused on the fact that, both health and disease are the result of the patient’s own actions, willpower and set of sustained efforts that the patient themselves must make to maintain an optimal state of health according to their conditions and characteristics (Kurtović et al., 2018).

However, the perception of disease as a construct has been widely studied in the field of health, coincident with the fact that patients construct different representations to make sense of the experience of disease to develop coping responses (Hagger and Orbell, 2003). This is a process in which beliefs are formed related to the nature of the disease, the course of time, the consequences or personal impact, possible causes and control/cure relationship, and this approach provides opportunities to help converge the beliefs of the physician and patient in order to achieve significant positive influences including not only the improvement of physical health, but also mental health conditions, since the dissonance between treatment and patient beliefs may increase the symptoms of anxiety and depression undermining their mental health (Broadbent et al., 2009; Morgan et al., 2014).

Among older adults that have psychiatric pharmacological treatment the greater perception of disease suggests a need to inquire about the moderating effect that this condition may have on self-care behaviors, given that although “severity” predicts the presence of self-care behaviors. It has also been identified that a poor emotional state and the belief that the disease is out of control, does not always translate into greater self-management (Riegel et al., 2009; Goodman et al., 2013).

Another relevant characteristic in the results of this research focused on the circumstances of the regime to which the adults belonged. The participants within the contributory regime presented lower scores in severity of disease consequences compared to the older adults benefited from health services corresponding to the subsidized regime. In this sense, it is necessary to contextualize that the subsidized regime is a governmental mechanism aimed at the poorest population of the country that do not have the capacity to pay and access health services through a subsidy offered by the state, while the contributory regime consists of a health system aimed at the population that has the capacity to pay and the monthly income to do so.

This makes sense given that the population belonging to the subsidized regime has lower economic income and socioeconomic characteristics of high vulnerability. Thus, this measure of “severity” of the disease proves that those older adults with better incomes tend to score lower in this dimension perceiving that the consequences of the disease may be less catastrophic compared to the group of adults who are more economically vulnerable and whose consequences may translate into difficulties accessing treatments or an inability to obtain some kind of income for their subsistence, contributing to a cyclical process in which subjective well-being and the perception of mental health are reduced, also undermining their physical health (April et al., 2012).

A fundamental feature in the study of health-disease processes in older adults is associated to the risk and protective factors that contribute to the development of the disease in different directions and in which early intervention could modify the adverse circumstances of the individual. Along these lines, the findings of this study suggest the presence of “fatigue severity” as a probable risk factor for physical function, since as scores of the former increase, so do scores of the latter. These data are consistent with the existing literature, which suggests a significant relationship between the feeling of fatigue often experienced by people diagnosed with a chronic disease and a lower presence of caregiving behaviors (Stadje et al., 2016; Son, 2019).

It is important to emphasize the influence of health- associated quality of life on the *“physical function”* scores found in this study, an issue that shows a need to discover the multiple ways in which this construct manifests itself and which can be translated into healthy habits and lifestyles that contribute to an optimal state of health for the individual. In general terms, health-related quality of life refers to the subjective evaluation of the interaction between current state of health, care, and promotional activities for maintaining general functioning and according to the data it seems that this assessment of the participants helps by increasing the physical activity among adults, which is linked to habits that promote actions for caring for one’s health (Urzua, 2010).

Finally, evidence was found that self-efficacy and emotional self-regulation positively influence health-related quality of life. Self-efficacy has been studied in the field of health to refer to the perception that people have of their abilities to perform a task, which can lead to people adopting behaviors that care for their health. The results in this field coincide with what has been proposed by different researchers who state that this construct is fundamental to monitoring chronic diseases internally in the long term (Senécal et al., 2000; Fathi et al., 2011).

Conversely, emotional self-regulation suggests that individuals who learn to regulate their emotions have a greater capacity to attenuate, maintain, increase or modify behaviors according to their goals and the conditions of the context in which they are in showing better physical health indicators (Jenaabadi et al., 2015; Hoorelbeke et al., 2016), an issue that coincides with what was found in this study and this highlights the need for carrying out preventive actions related to this variable.

The set of results found, supports the results of different investigations that have found information in the same vein, demonstrating that there is a need to carry out health promotion and prevention strategies aimed at the older adult population that take into consideration factors such as socioeconomic characteristics, beliefs, and perceptions of the disease, as well as individual conditions for the functional coping of chronic disease.

## 5. Limitations and strengths

We had as a limitation in the study that it was not possible to establish representativeness of the sample, because in Colombia there is no reliable and consolidated information on the exact number of people who suffer from chronic diseases.

This is the first study to be developed in Colombia, evaluating self-efficacy, illness perception, emotional regulation and fatigue on the quality of life associated with the health of older adults. Generating new knowledge for this country.

## 6. Conclusion

The results of this study show that older adults that have psychiatric medication tend to present lower scores in personal control and a greater perception of disease, which exacerbates their emotional state, and they perceive the disease’s effects to be a large burden, an issue that should be studied in depth considering that the perception of disease is closely associated to the emotional state of the subject.

Furthermore, it was found that socioeconomic status significantly increased the scores in association to the consequences of the disease, which denotes a need for these features to be addressed given that negative perceptions about the disease’s effects contribute to increased depression and anxiety among patients with chronic disease.

Another relevant aspect was to identify the perception of illness and, to a greater extent, the severity of fatigue as factors that could have a negative impact on the physical function of the participants, which contributed significantly to the presence of low scores in physical activity.

Finally, it was found that self-efficacy and emotional self-regulation can increase the positive perception of health-related quality of life.

## Data availability statement

The original contributions presented in the study are included in the article/supplementary material, further inquiries can be directed to the corresponding author.

## Ethics statement

The studies involving human participants were reviewed and approved by Comité de Ética de la Clínica General del Norte en Barranquilla-Colombia. The patients/participants provided their written informed consent to participate in this study.

## Author contributions

LC-T and SV had full access to all study data and assumed responsibility for the integrity of the data and the accuracy of the analysis. LC-T and SV: concept and design. LC-T, NG, ÁB-N, and JH-L: acquisition, analysis, or interpretation of data. LC-T, SV, MB, and NG: the drafting of the manuscript. LC-T, SV, ÁB-N, NG, JM, LA, GR-C, SC-C, VB, JH-L, CR, MB, and JÁ: critical revision of the manuscript for important intellectual content. JH-L and VB: Statistical analysis. LC-T and VB: acquisition of funding. LC-T, SV, CR, GR-C, SC-C, JM, LA, and JÁ: technical, administrative, or material support. LC-T and SV: supervision. All authors contributed to the article and approved the submitted version.

## Funding

The study was funded by the Fundación Universitaria del Área Andina, Universidad del Sinú, Universidad Popular del Cesar, Universidad Simón Bolívar and Clínica General del Norte.

## Conflict of interest

The authors declare that the research was conducted in the absence of any commercial or financial relationships that could be construed as a potential conflict of interest.

## Publisher’s note

All claims expressed in this article are solely those of the authors and do not necessarily represent those of their affiliated organizations, or those of the publisher, the editors and the reviewers. Any product that may be evaluated in this article, or claim that may be made by its manufacturer, is not guaranteed or endorsed by the publisher.
